# Long-Term Results of Concurrent Chemoradiotherapy for Advanced N2-3 Stage Nasopharyngeal Carcinoma

**DOI:** 10.1371/journal.pone.0137383

**Published:** 2015-09-14

**Authors:** Li Yin, Xiu-Hua Bian, Xue Wang, Meng Chen, Jing Wu, Jian-Hua Xu, Pu-Dong Qian, Wen-Jie Guo, Xue-Song Jiang, Huan-Feng Zhu, Jia-Jia Gu, Jian-Feng Wu, Ye-wei Zhang, Xia He

**Affiliations:** 1 Department of Radiation Oncology, Nanjing Medical University Affiliated Jiangsu Cancer Hospital & Jiangsu Institute of Cancer Research, Nanjing, P. R. China; 2 Department of Radiation Oncology, Jilin Cancer Hospital, Changchun, P. R. China; 3 Department of Hepatobiliary and Pancreatic Surgery, Nanjing Medical University Affiliated Jiangsu Cancer Hospital & Jiangsu Institute of Cancer Research, Nanjing, P. R. China; Northwestern University Feinberg School of Medicine, UNITED STATES

## Abstract

**Background:**

N-stage is related to distant metastasis in nasopharyngeal carcinoma (NPC) patients. The purpose of this study was to evaluate the efficacy and toxicity of different nedaplatin-based chemotherapy regimens in advanced N2-3 stage NPC patients treated with intensity modulated radiation therapy (IMRT).

**Patients and Methods:**

Between April 2005 and December 2009, a total of 128 patients with N2-3 advanced NPC were retrospectively analyzed. Patients were treated with IMRT concurrent with 2 cycles of chemotherapy consisting of either nedaplatin plus paclitaxel (NP group, n = 67) or nedaplatin plus fluorouracil and paclitaxel (NFP group, n = 61). Two to four cycles of adjuvant chemotherapy were then administered every 21 days following concurrent chemoradiotherapy.

**Results:**

With a median follow-up of 60 months, the 5-year overall survival (OS), progression-free survival (PFS), local-regional recurrence-free survival (LRRFS), and distant metastasis-free survival (DMFS) for all patients were 81.4%, 71.5%, 87.8% and 82.0%, respectively. No significant difference in PFS (66.6% vs. 76.7%, *P* = 0.212) and LRRFS rates (89.0% vs. 86.3%, *P* = 0.664) was observed between the NP and NFP groups. The 5-year OS (75.4% vs. 88.5%, *P* = 0.046) and DMFS (75.1% vs. 89.0%, *P* = 0.042) rate were superior in the NFP group compared with the NP group. The NFP group had a higher incidence of grade 3–4 acute toxicities including bone marrow suppression (leukopenia: χ2 = 3.935, *P* = 0.047; anemia: χ2 = 9.760, *P* = 0.002; thrombocytopenia: χ2 = 8.821, *P* = 0.003), and both liver and renal dysfunction (χ^2^ = 5.206, *P* = 0.023) compared with the NP group. Late toxicities were moderate and no difference was observed between the two groups.

**Conclusion:**

IMRT concurrent with nedaplatin-based chemotherapy is an advocated regimen for patients with advanced N2-3 stage NPC. Patients with advanced N2-3 stage may be better candidates for the NFP regimen although this regimen was associated with a high acute toxicity rate.

## Introduction

Nasopharyngeal carcinoma (NPC) is endemic in south-east Asia, parts of Africa and southern China. Radiotherapy plays a key role in the treatment of all stages of NPC without distant metastasis [1–2]. Approximately 70% of newly diagnosed NPC patients present with stage III or IV disease, and are prone to local-regional recurrence or distant metastases after radiotherapy alone [3]. Intensity-modulated radiotherapy (IMRT) is a major breakthrough in the treatment of NPC. IMRT delivers a higher dose to the tumor, while minimizing the dose to surrounding normal tissues. Previous studies have confirmed that IMRT changed the failure pattern of NPC from local recurrence and distant metastasis to predominantly distant metastasis [4,5].

It is also well known that N-stage is related to distant metastasis in NPC patients. Xu et al. reviewed 181 NPC patients and found that the 3-year distant metastasis-free survival rate (DMFS) was 89.6%, 75.7%, and 76.3% for stageT3-4N0-1, T1-2N2-3, and T3-4 N2-3 patients, respectively. Cox proportional hazards regression analysis showed that advanced N-stage was an independent risk factor for distant metastasis (*P* = 0.033) [6]. Therefore, it is necessary to explore more effective combined chemoradiotherapy strategies to improve the outcomes of patients with advanced N-stage NPC.

The cisplatin-based Intergroup-0099 regimen resulted in a significantly increased toxicity rate. According to the results of previous research [7–10], nedaplatin-based regimens achieved comparable survival, were well tolerated and had lower toxicity than cisplatin-based regimens in the treatment of head and neck cancer, esophageal cancer, uterine cervix cancer and NPC. Therefore, we retrospectively analyzed the long-term outcomes of advanced N-stage NPC patients treated with concurrent IMRT and nedaplatin-based chemotherapy, followed by adjuvant chemotherapy, and compared the efficacy and toxicity of different nedaplatin-based chemotherapy regimens in these patients.

## Patients and Methods

### Patients

Between April 2005 and December 2009, 128 pathologically confirmed N2-3 stage NPC patients without distant metastasis in Jiangsu Cancer Hospital were enrolled in this retrospective analysis. All patients had no previous treatment history and underwent definitive IMRT at our hospital. Initial workup included clinical and laboratory examinations, hematologic and biochemistry profiles, fiberoptic endoscope examination of the nasopharynx, magnetic resonance imaging (MRI) and contrast-enhanced computed tomography (CT) of the head and neck to evaluate the extent of the primary tumor and regional lymph nodes. Bone scintigraphy, chest radiography or contrast-CT, and ultrasonography of the abdominal region were performed to exclude distant metastasis. Stages were assigned according to the 6th edition of the American Joint Committee on Cancer (AJCC TNM 2002) staging system [11].

### IMRT

**Fig 1:**
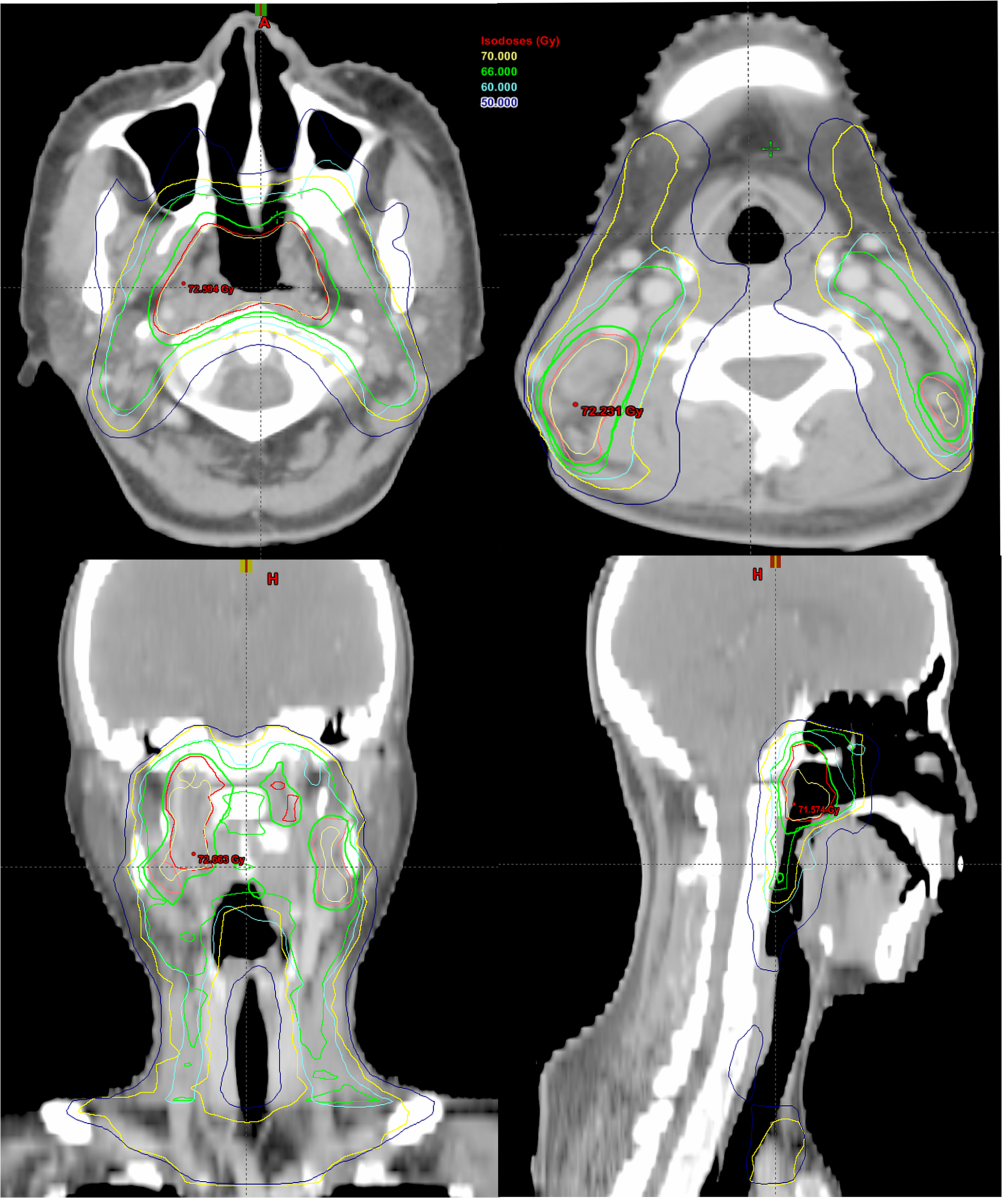
Target volume contouring in 3-dimensional intensity-modulated radiotherapy plan of nasopharyngeal carcinoma. (Red line represents GTV1, pink line represents GTV2, green line represents CTV1 and yellow line represents CTV2; Isodose levels: 50 Gy in blue line, 60 Gy in cyan line, 66 Gy in green line and 70 Gy in yellow line).

Patients received definitive IMRT with 6 MV X-rays as previously reported [6]. Briefly, gross tumor volume (GTV) 1 included the primary tumor and metastatic retropharyngeal lymph nodes found on clinical and imaging examination. Metastatic cervical lymph nodes were defined as GTV2. The high-risk region was defined as clinical target volume (CTV)1 which included the whole nasopharyngeal cavity, GTV1, GTV2 with a margin of 5 to 15 mm, and levels II and III cervical lymphatic drainage region. Low risk area was defined as CTV2 which encompassed CTV1 with a margin of 3 to 5 mm, the lower neck, and the supraclavicular lymphatic drainage region. A total dose of 67.6–78.0 Gy/31–35 F was prescribed to GTV1, 65.0–85.0 Gy/32–39 F to GTV2, 56.0–60 Gy/30 F to CTV1 and 50 Gy/30 F to CTV2, respectively. All patients were irradiated with 1 fraction daily, 5 days per week. An example of the three-dimensional dose distribution described is shown in Fig 1.

### Chemotherapy

During the period of concurrent chemoradiotherapy, 61 of 128 patients (47.7%) received the NFP regimen, which consisted of paclitaxel 135–175 mg/m^2^ per cycle on day 1, fluorouracil 500–600 mg/m^2^ on days 1 to 5, and nedaplatin with a total dosage of 80–100 mg/m^2^ per cycle evenly administered on days 1 to 3. The remaining 67 patients (52.3%) were treated with the NP regimen, which consisted of paclitaxel 135–175 mg/m^2^ per cycle on day 1 and nedaplatin with a total dosage of 80 to 100 mg/m^2^ per cycle evenly administered on days 2 to 4. Concurrent chemotherapy was limited to two cycles with an interval of 3–4 weeks. Adjuvant chemotherapy with the NP or NFP regimen after approximately 4 weeks’ restoration of combined therapy was given repeatedly every 3 to 4 weeks according to the performance status. The chemotherapy during the whole treatment period was limited to no more than six cycles in total.

Complete blood count, liver and renal function were tested before each chemotherapy cycle. Dose modification was carried out by the attending radiation oncologists according to the toxicity level [10]. Granulocyte colony-stimulating factor (G-CSF) and recombinant erythropoietin (EPO) were not administered unless the white blood cell count dropped to less than 3.5× 10^9^/L and hemoglobin decreased to less than 100 g/L, respectively. Antibiotics were administered to patients with neutropenic fever. Platelet transfusion with or without administration of interleukin 11 or recombinant thrombopoietin was administered when the platelet count was less than 25 ×10^9^/L. Red blood cell transfusions were administered when hemoglobin decreased to less than 70 g/L.

### Follow-up and treatment assessment

Toxicity was assessed using the National Cancer Institute Common Terminology Criteria for Adverse Events (CTCAE, version 3.0). Regular follow-up visits were performed at our institution. Patients were evaluated for disease control, survival and toxicity at three-month intervals for the first two years, at 6-month intervals between the third and fifth years, and 1 year intervals thereafter. Each follow-up visit included a clinical physical examination, nasopharyngoscopy, ultrasonography of the abdomen and chest X-ray. A CT scan or MRI of the head and neck region was conducted every 3 to 6 months for the first 2 years and then yearly from year 3. In patients with evidence of local-regional recurrence or distant metastasis, additional examination or imaging modalities were performed to confirm or exclude disease progression at the discretion of the treating physician.

### Statistical analysis

Statistical analysis was performed using SPSS 20.0 (International Business Machines Corp, Armonk, NY, USA). Overall survival (OS), progression-free survival (PFS), local-regional recurrence-free survival (LRRFS), and distant metastasis-free survival (DMFS) were defined as follows: OS, survival during the follow-up period; PFS, survival without local-regional failure or distant metastasis; LRRFS, survival without recurrence in the nasopharynx or cervical lymph nodes; and DMFS, survival without distant metastasis. OS, PFS, LRRFS and DMFS rates were determined using the Kaplan-Meier method. Differences between survival curves were compared using log-rank tests. Univariate and multivariate survival analyses were performed using the Cox proportional hazards regression model. Statistical significance was accepted when the *P* value was <0.05.

### Ethical statement

This retrospective study was approved by the Jiangsu Cancer hospital review board and was conducted in accordance with the ethical guidelines of the Declaration of Helsinki.

## Results

### Patient characteristics

The median duration of follow-up was 60.0 months (range, 11.0–109.0 months). A total of 128 NPC patients with advanced N2-3 stage were enrolled in this retrospective study (67 in the NP group and 61 in the NFP group). The mean age at the time of NPC diagnosis was 49.5 ± 12.9 years. There were 91 males and 37 females. Stage distribution was as follows: stage III, 76; stage IV_a_, 27; and stage IV_b_, 25. Patient characteristics and treatment facts of the 2 groups are shown in Table 1.

**Table 1:** Patient characteristics. Abbreviations: AJCC, American Joint Committee on Cancer; NP, nedaplatin plus paclitaxel; NFP, nedaplatin, paclitaxel plus fluorouracil

Characteristic	NP		NFP	
	No. of patients	(%)	No. of patients	(%)
**Total**	67		61	
**Gender**				
Male	47	70.1	44	72.1
Female	20	29.9	17	27.9
**Age (years)**				
≤50	35	52.2	34	55.7
>50	32	47.8	27	44.3
**AJCC staging** *				
Ⅲ	42	62.7	34	55.7
Ⅳa	12	17.9	15	24.6
Ⅳb	13	19.4	12	19.7
**AJCC T classification** *				
T1	5	7.5	4	6.6
T2	17	25.4	9	14.8
T3	32	47.8	31	50.8
T4	13	19.4	17	27.9
**AJCC N classification** *				
N2	54	80.6	49	80.3
N3	13	19.4	12	19.7
Adjuvant chemotherapy				
No	3	4.5	7	11.5
1–2 cycles	40	59.7	43	70.5
3–4 cycles	24	35.8	11	18.0

* **Defined by the criteria of the AJCC 2002 staging system [11].**

### Survival

**Fig 2:**
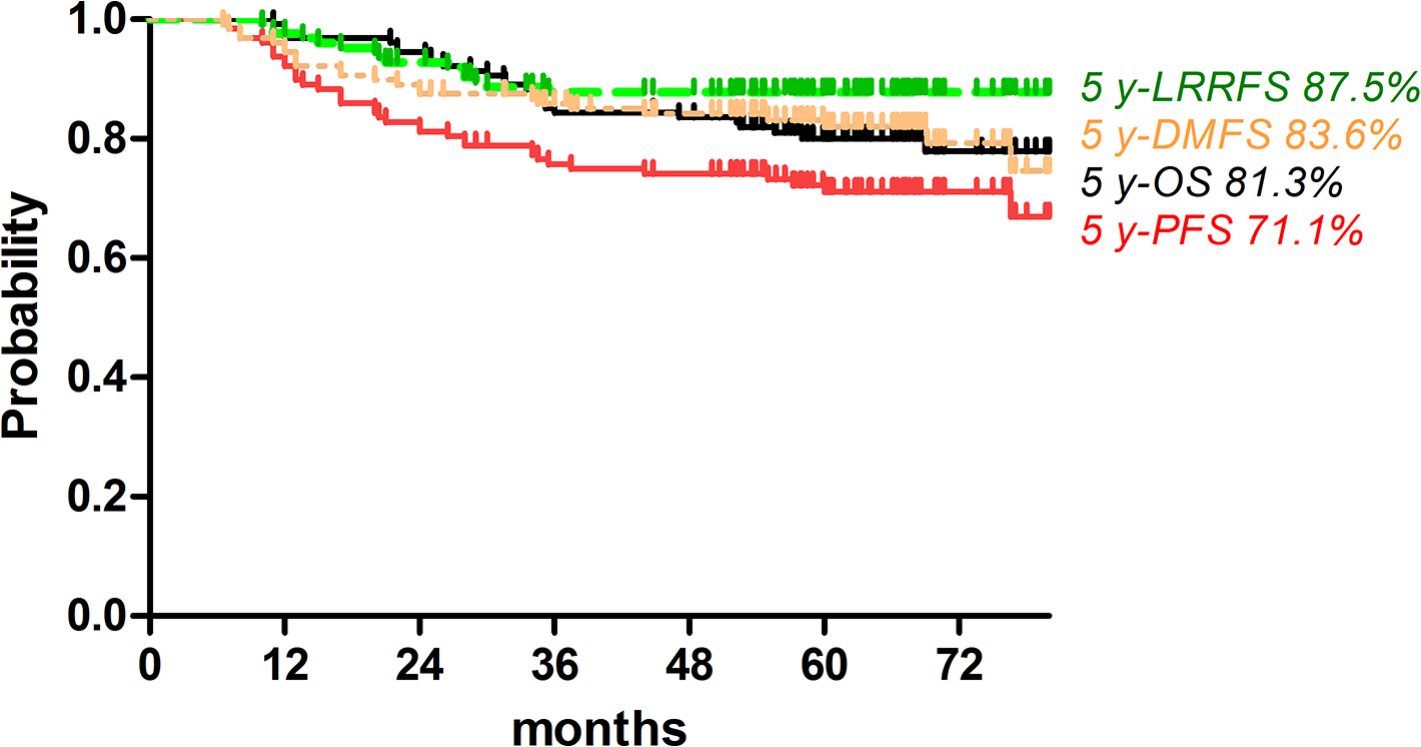
Kaplan-Meier curves of overall survival (OS), progression-free survival (PFS), locoregional recurrence-free survival (LRRFS) and distant metastasis-free survival (DMFS) for 128 patients with advanced N2-3 stage NPC.

**Fig 3:**
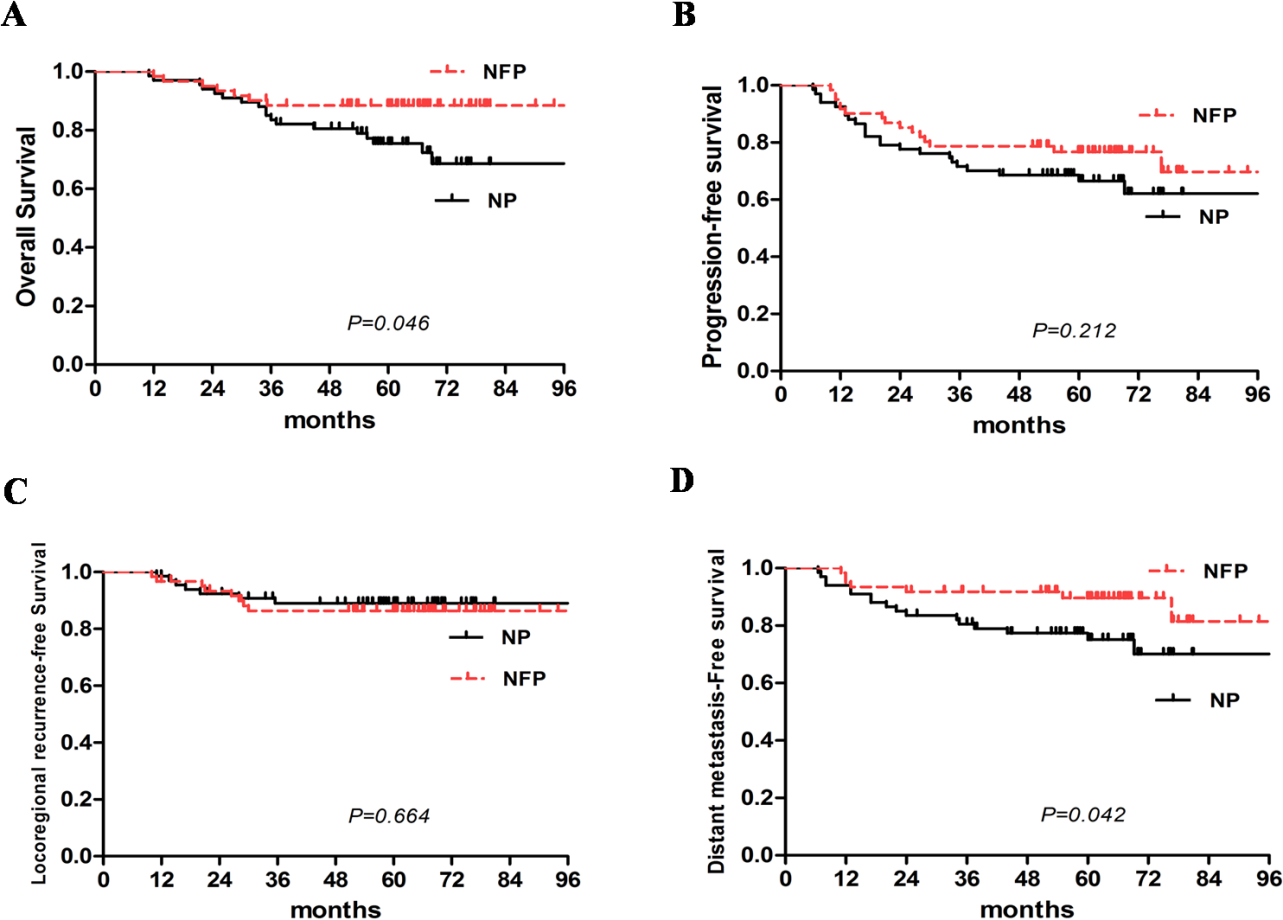
5-year OS, PFS, LRRFS and DMFS were 75.4% vs. 88.5%, 66.6% vs. 76.7%, 89.0% vs.86.3%, and 75.1% vs. 89.0% for the NP and NFP groups, respectively. A and D. Advanced N2-3 stage NPC patients in the concurrent NFP group had a significantly superior OS and DMFS (Log Rank test, OS: χ2 = 3.973, *P* = 0.046; DMFS: χ2 = 4.130, *P* = 0.042). B and C. No significant difference in PFS and LRRFS were seen during the observation period. (Log Rank test, PFS: χ2 = 1.560, *P* = 0.212; LRRFS: χ2 = 0.189, *P* = 0.664).

For all patients, the 5-year OS, PFS, LRRFS and DMFS were 81.4%, 71.5%, 87.8% and 82.0%, respectively (Fig 2). There were no significant differences in the 5-year PFS (66.6% vs. 76.7%, *P* = 0.212) and LRRFS rates (89.0% vs. 86.3%, *P* = 0.664) between the NP and NFP groups. The 5-year OS (75.4% vs. 88.5%, *P* = 0.046) and DMFS rate (75.1% vs. 89.0%, *P* = 0.042) were inferior in the NP group compared with the NFP group (Fig 3A–3D).

### Prognostic factors

We performed univariate and multivariate analyses to assess the value of various potential prognostic factors on predicting OS, PFS, LRRFS and DMFS. The following factors were included as covariates: gender, age, AJCC staging (Ⅲ vs. Ⅳ), AJCC T classification (T_1–2_ vs. T_3–4_), and AJCC N classification (N_2_ vs. N_3_), concurrent chemotherapy regimen (NP vs. NFP), radiation dose to primary lesion and positive lymph nodes (≤70 Gy vs.>70 Gy) and adjuvant chemotherapy cycles. Univariate analysis by log-rank test showed that AJCC staging was significantly associated with OS, PFS, and DMFS (AJCC staging: *P* = 0.001, *P* = 0.001, and *P* = 0.002, respectively). AJCC T classification was associated with LRRFS (*P* = 0.019) and AJCC N classification was associated with PFS and DMFS (*P* = 0.034 and *P* = 0.002). In addition, gender was also significantly associated with PFS and DMFS (*P* = 0.038 and *P* = 0.018) (S1 Table).

Multivariate analysis was performed using the Cox progression model to assess the independent risk factors for survival rates and the results are listed in Table 2. Adjusted by age, adjuvant chemotherapy and radiation dose, the results showed that AJCC staging was an independent prognostic predictor of OS, PFS, and DMFS (*P* = 0.01; *P* = 0.00; *P* = 0.02, respectively). Chemotherapy regimen independently influenced OS and DMFS (*P* = 0.03 and *P* = 0.01). In addition, gender was an independent prognostic predictor of PFS (*P* = 0.03) and DMFS (*P* = 0.02).

**Table 2:** Multivariate prognostic factor analyses for various clinical endpoints. Abbreviation: HR: hazard ratio; CI: confidence interval.

Endpoint	Characteristics	HR (95% CI)	*P* value
OS	Gender	0.73(0.30–1.76)	0.48
	AJCC staging	6.63(1.53–28.70)	0.01
	T classification	0.79(0.38–1.62)	0.51
	N classification	0.51(0.12–2.18)	0.36
	Chemotherapy	0.36(0.15–0.89)	0.03
PFS	Gender	0.37(0.15–0.89)	0.03
	AJCC staging	5.35(1.76–16.27)	0.00
	T classification	0.78(0.46–1.31)	0.34
	N classification	0.60(0.19–1.89)	0.39
	Chemotherapy	0.58(0.30–1.14)	0.11
LRRFS	Gender	0.74(0.23–2.45)	0.62
	AJCC staging	4.02 (0.67–24.02)	0.13
	T classification	0.92(0.33–2.57)	0.87
	N classification	0.27(0.03–2.31)	0.23
	Chemotherapy	1.33(0.45–3.94)	0.61
DMFS	Gender	0.18(0.04–0.78)	0.02
	AJCC staging	5.39(1.26–23.04)	0.02
	T classification	0.77(0.41–1.44)	0.41
	N classification	0.93(0.22–3.96)	0.93
	Chemotherapy	0.32(0.13–0.78)	0.01

### Toxic effects

Acute toxicities were assessed in all 128 patients and are summarized in Table 3. The most common acute toxicities were hematological toxicity and mucositis. For patients who received the NP regimen, the incidence of grade 3–4 hematologic toxicities (leukopenia, anemia or thrombocytopenia), oral mucositis, vomiting and both liver and renal function abnormalities (ALT elevation or sCr elevation) was 44.8%, 14.9%, 25.4%, 31.3%, 31.3% and 20.9%, respectively. For patients who received the NFP regimen, these values were 62.3%, 39.3%, 50.8%, 39.3%, 45.9% and 39.3%, respectively. The NFP group had a significantly higher incidence of moderate-to-severe toxicities, including grade 3–4 bone marrow suppression (leukopenia: χ^2^ = 3.935, *P* = 0.047; anemia: χ^2^ = 9.760, *P* = 0.002; thrombocytopenia: χ2 = 8.821, *P* = 0.003) and both liver and renal dysfunction (χ^2^ = 5.206, *P* = 0.023). For the sake of bone marrow suppression, G-CSF and EPO were used in 44 and 17 patients in the NP group, respectively. The number of patients in the NFP group receiving G-CSF and EPO were 52 and 35, respectively. Vomiting (χ^2^ = 2.864, *P* = 0.091) and oral space mucositis (χ^2^ = 0.897, *P* = 0.344) were common in the NFP group, however, the differences were not significant.

**Table 3:** Severe acute toxicities based on the CTCAE3.0 grading system. Abbreviations: CTCAE, Common Terminology Criteria for Adverse Events.

Acute toxicities (Grade 3/4 acute toxicities)	No. of the NP group (%)	No. of the NFP group (%)	*P* value
Leukopenia	30(44.8)	38(62.3)	0.047
Anemia	10(14.9)	24(39.3)	0.002
Thrombocytopenia	17(25.4)	31(50.8)	0.003
Liver/ Renal dysfunction	14(20.9)	24(39.3)	0.023
Oral mucositis	21(31.3)	24(39.3)	0.344
Vomiting	21(31.3)	28(45.9)	0.091

The main documented late toxicities in patients treated with IMRT and concurrent NP or NFP chemotherapy who survived for more than 2 years were fibrosis (skin and subcutaneous tissue of the neck), xerostomia, hearing loss and visual impairment. Other late toxicities observed included trismus, radiation-induced brain injury (temporal necrosis, cranial nerve palsy) and subclinical hypothyroidism. No patient had grade 4 or 5 late toxicity. No differences were observed for grade 3 late toxicities (Table 4).

**Table 4:** Treatment-related late toxicities.

Late toxicities (Grade 3)	No. of the NP group (%)	No. of the NFP group (%)	*P* value
Xerostomia	1(1.5)	0(0)	0.338
Fibrosis	4(6.0)	4(6.6)	0.891
Hearing or vision impairment	7(10.4)	6(9.8)	0.909
Hypothyroidism	1(1.5)	1(1.6)	0.991
Trismus	6(9.0)	5 (8.2)	0.878
Radiation-induced brain injury	2(3.0)	3(4.9)	0.573

## Discussion

Concurrent chemoradiotherapy (CCRT) can achieve significantly better tumor control than radiotherapy alone and it is the standard treatment for locoregionally advanced NPC based on large randomized phase Ⅲ trials and a meta-analysis [12,13]. Although it is generally accepted that platinum monotherapy or platinum-based doublet chemotherapy has been the standard of care for years, the combination of docetaxel, platinum, and fluorouracil has also been evaluated [14–17] and the toxicity is manageable in patients with head and neck squamous cell carcinomas. However, the additive value or the optimal combination schedule of chemotherapy and radiotherapy is largely unknown at present.

With optimized local treatment achieved with IMRT, locoregional control has been markedly improved in NPC [18]. The main cause of failure in NPC is distant metastasis following IMRT [19]. N-stage is a prognostic factor in NPC patients, and irrespective of the radiation technique used, advanced N-stage patients have a high rate of distant metastasis [4,18,20]. Chemotherapy plays an important role in reducing the probability of metastasis. It is reasonable to consider more intensive modifications of chemotherapy in addition to IMRT for advanced N stage disease. To our knowledge, there is no published randomized phase III study confirming the value of chemotherapy in NPC patients treated with IMRT. Our study is the first to compare concurrent chemotherapy regimens in patients with advanced N stage NPC.

Recently, Wu et al. [21] prospectively evaluated the long-term outcome in III–IVb NPC patients treated with IMRT and concurrent cisplatin (80–100 mg/m^2^). The 5-year OS, LRFS, RRFS, and DMFS were 78.4%, 86.8%, 88.4%, and 78.0%, respectively. In addition, Sun et al. [22] reported that the 5-year OS, RFS, DMFS and PFS in advanced N-stage NPC patients treated with chemoradiotherapy (IMRT) were 73.1%, 91.3%, 68.5% and 63.8%, respectively. The 5-year OS, PFS and DMFS in our series were favorable and comparable, which demonstrated the efficacy of nedaplatin-based chemoradiotherapy in advanced N stage NPC. On the other hand, no significant difference in 5-year PFS and LRRFS rates was observed between the two groups, however, the 5-year OS and DMFS rates were superior in the NFP group compared with the NP group (75.4% vs. 88.5%, 75.1% vs. 89.0%, respectively). The increase in DMFS translate into a substantial improvement in OS. This indicates that aggressive chemotherapy could reduce the risk of metastasis. According to our results, aggressive chemotherapy was more effective in eradicating distant tumors in advanced N-stage NPC patients. TAX324, a randomized phase Ⅲ trial conducted in advanced head and neck cancer patients [23], has shown DMFS advantages due to multi-agent intensive chemotherapy, which also indicated that a more effective regimen of chemotherapy should be considered to overcome the higher incidence of distant metastasis in patients with advanced N stage tumors. Table 5 summarizes the OS and DMFS in advanced N stage NPC patients [4,18,20,22,24–25]. Despite the retrospective nature and limited patient number in our analysis, the outcome of our advanced N stage NPC patients treated with IMRT and concurrent NP or NFP chemotherapy with the addition of adjuvant chemotherapy was comparable to the results published by other groups. In addition, EPO and G-CSF were used when patients had bone marrow suppression. Interestingly, the phase III randomized RTOG trial 9903 showed that administration of EPO in head and neck cancer patients is not helpful and may be harmful[26]. However, in the present study, although more patients in the NFP group than the NF group received EPO treatment, the survival results were still better in the NFP group than the NF group. This discrepancy may be due to different biology behavior and treatment response between NPC and head and neck cancer. Future further exploration in a randomized setting warrants for resolving this issue.

**Table 5:** OS and DMFS of patients with advanced N stage NPC treated with chemoradiotherapy. Abbreviations: RT, radiotherapy; 2D-RT, two-dimensional RT; IMRT, intensity-modulated radiotherapy.

Year	Author	No.	RT	OS (%)	DMFS (%)
2001	Ma et al.[25]	621	2D-RT	-	5-year
					N2:62
					N3:51
2005	Leung et al.[24]	1070	2D-RT	5-year	5-year
				N2:60.3	N2:71.6
				N3:40.2(a) and 48.4 (b)	N3:66.9(a) and 52.1(b)
2010	Wong et al.[4]	175	IMRT	-	3-year
					N2:76.6
					N3:67.3
2011	Lai et al.[18]	1276	2D-RT	-	5-year
					N2:75.1
					N3:65.4
			IMRT		N2:80.5
					N3:60.9
2013	Sun et al.[22]	198	IMRT	5-year	5-year
				N2:71.0	N2:68.5
				N3:55.7	N3: 50.1
2014	Sun et al.[20]	868	IMRT	5-year	5-year
				Ⅲ:83.6	N2:73.7
				Ⅳ:70.5	N3:62.1

The dose-limiting toxicity of nedaplatin is bone marrow suppression [27]. However, bone marrow suppression was reversible and no treatment-related deaths were observed in the present study. In addition, the incidence of severe gastrointestinal toxicity (grade ≥3) was 27.3–32.1% in cisplatin-based CCRT [28,29]. The nedaplatin-based CCRT study showed a lower incidence of grade ≥2 vomiting [10]. As fluorouracil has additive gastrointestinal toxicity with cisplatin, concurrent cisplatin plus fluorouracil with radiotherapy produced a relatively high incidence of moderate to severe gastrointestinal toxicity in the treatment of locoregionally advanced NPC [5,28]. Nedaplatin causes less gastrointestinal toxicity and is suitable for combination with other chemotherapy agents. When fluorouracil was added to nedaplatin and paclitaxel, the incidence of vomiting significantly increased in the NFP group. Acute mucositis was common in the present study (31.3% vs. 50.8%). However, this toxicity was also common (61%) in studies on cisplatin regimens [29]. Thus, oncologists should be aware of these common adverse events, and symptomatic treatment should be offered to patients, which may have an impact on patient compliance to therapy and quality of life. Moreover, the nedaplatin regimen took less time to administer as there was no pre- and post-hydration. Furthermore, the most common late toxicity was grade 1–2 neck fibrosis, xerostomia, hearing impairment and vision loss, similar to other reports [22]. No differences were observed in the two groups regarding grade 3 late toxicities.

Our results demonstrated that AJCC staging was significantly associated with OS, PFS, and DMFS. Chemotherapy independently influenced OS and DMFS. These results suggest that aggressive and intensive systemic therapy for advanced N stage NPC should be considered. In addition, gender was also significantly associated with PFS and DMFS in our study, which might have been associated with the disparity of gender in the enrolled patients.

There are several limitations in the current study, including the retrospective nature of the study design, the wide variation in radiation dose and fractionation, which could affect the outcome. Nevertheless, our report is noteworthy because this is the first study to evaluate concurrent nedaplatin-based chemotherapy for advanced N2-3 stage NPC treated by IMRT.

In summary, our data indicate the benefit of concurrent NP or NFP chemotherapy with IMRT in patients with advanced N stage NPC, especially the NFP regimen which resulted in a good 5-year OS and DMFS. This regimen may be considered as an alternative therapeutic regimen for this patient group even though it is associated with a high acute toxicity rate. Further investigation in the prospective setting is warranted to explore the role of concurrent NFP and adjuvant chemotherapy in IMRT treatment of advanced N stage NPC.

## Supporting Information

S1 Data Original data used for all statistical analysis in this article. (SAV)

S1 Table Univariate prognostic factor analyses for various clinical endpoints. (DOCX)

## References

[1] Lo KW, Chung GT, To KF. Deciphering the molecular genetic basis of NPC through molecular, cytogenetic, and epigenetic approaches. Seminars in cancer biology. 2012;22(2):79–86. 10.1016/j.semcancer.2011.12.011 . 22245473

[2] Ji X, Xie C, Hu D, Fan X, Zhou Y, Zheng Y. Survival benefit of adding chemotherapy to intensity modulated radiation in patients with locoregionally advanced nasopharyngeal carcinoma. PloS one. 2013;8(2):e56208 10.1371/journal.pone.0056208 23441169 PMC3575472

[3] Zhang L, Zhao C, Ghimire B, Hong MH, Liu Q, Zhang Y, et al The role of concurrent chemoradiotherapy in the treatment of locoregionally advanced nasopharyngeal carcinoma among endemic population: a meta-analysis of the phase III randomized trials. BMC cancer. 2010;10:558 10.1186/1471-2407-10-558 20950416 PMC2970609

[4] Wong FC, Ng AW, Lee VH, Lui CM, Yuen KK, Sze WK, et al Whole-field simultaneous integrated-boost intensity-modulated radiotherapy for patients with nasopharyngeal carcinoma. International journal of radiation oncology, biology, physics. 2010;76(1):138–45. 10.1016/j.ijrobp.2009.01.084 . 19646824

[5] Lee N, Harris J, Garden AS, Straube W, Glisson B, Xia P, et al Intensity-modulated radiation therapy with or without chemotherapy for nasopharyngeal carcinoma: radiation therapy oncology group phase II trial 0225. Journal of clinical oncology: official journal of the American Society of Clinical Oncology. 2009;27(22):3684–90. 10.1200/JCO.2008.19.9109 19564532 PMC2720082

[6] Xu JH, Guo WJ, Bian XH, Wu JF, Jiang XS, Guo YS, et al A comparative study of locoregionally advanced nasopharyngeal carcinoma treated with intensity modulated irradiation and platinum-based chemotherapy. Cancer radiotherapie: journal de la Societe francaise de radiotherapie oncologique. 2013;17(4):297–303. 10.1016/j.canrad.2013.03.006 . 23849438

[7] Ohashi T, Ohnishi M, Tanahashi S, Murai M. Efficacy and toxicity of concurrent chemoradiotherapy with nedaplatin and S-1 for head and neck cancer. Japanese journal of clinical oncology. 2011;41(3):348–52. 10.1093/jjco/hyq196 . 21109512

[8] Shen ZT, Wu XH, Li B, Shen JS, Wang Z, Li J, et al Nedaplatin concurrent with three-dimensional conformal radiotherapy for treatment of locally advanced esophageal carcinoma. World journal of gastroenterology: WJG. 2013;19(48):9447–52. 10.3748/wjg.v19.i48.9447 24409075 PMC3882421

[9] Li XF, Li YH, Gao YN, Li CL, Yue HZ, Xu G, et al [Comparison of two different chemotherapy regimens for concurrent chemoradiotherapy in stage Ib2 to IVa squamous cell carcinoma of the uterine cervix]. Zhonghua fu chan ke za zhi. 2013;48(10):763–7. . 24406134

[10] Xu J, He X, Cheng K, Guo W, Bian X, Jiang X, et al Concurrent chemoradiotherapy with nedaplatin plus paclitaxel or fluorouracil for locoregionally advanced nasopharyngeal carcinoma: Survival and toxicity. Head & neck. 2014;36(10):1474–80. 10.1002/hed.23487 . 23996842

[11] Kalogera-Fountzila A, Karanikolas D, Katodritis N, Samantas E, Sarafopoulos A, Ikonomou I, et al Prognostic factors and significance of the revised 6th edition of the AJCC classification in patients with locally advanced nasopharyngeal carcinoma. Strahlentherapie und Onkologie: Organ der Deutschen Rontgengesellschaft [et al]. 2006;182(8):458–66. 10.1007/s00066-006-1538-4 . 16896592

[12] Langendijk JA, Leemans CR, Buter J, Berkhof J, Slotman BJ. The additional value of chemotherapy to radiotherapy in locally advanced nasopharyngeal carcinoma: a meta-analysis of the published literature. Journal of clinical oncology: official journal of the American Society of Clinical Oncology. 2004;22(22):4604–12. 10.1200/JCO.2004.10.074 . 15542811

[13] Baujat B, Audry H, Bourhis J, Chan AT, Onat H, Chua DT, et al Chemotherapy in locally advanced nasopharyngeal carcinoma: an individual patient data meta-analysis of eight randomized trials and 1753 patients. International journal of radiation oncology, biology, physics. 2006;64(1):47–56. 10.1016/j.ijrobp.2005.06.037 . 16377415

[14] Argiris A, Karamouzis MV. Empowering induction therapy for locally advanced head and neck cancer. Annals of oncology: official journal of the European Society for Medical Oncology / ESMO. 2011;22(4):773–81. 10.1093/annonc/mdq426 . 20864569

[15] Hui EP, Ma BB, Leung SF, King AD, Mo F, Kam MK, et al Randomized phase II trial of concurrent cisplatin-radiotherapy with or without neoadjuvant docetaxel and cisplatin in advanced nasopharyngeal carcinoma. Journal of clinical oncology: official journal of the American Society of Clinical Oncology. 2009;27(2):242–9. 10.1200/JCO.2008.18.1545 . 19064973

[16] Kong L, Zhang YW, Hu CS, Guo Y. Neoadjuvant chemotherapy followed by concurrent chemoradiation for locally advanced nasopharyngeal carcinoma. Chinese journal of cancer. 2010;29(5):551–5. . 20426907 10.5732/cjc.009.10518

[17] Bae WK, Hwang JE, Shim HJ, Cho SH, Lee JK, Lim SC, et al Phase II study of docetaxel, cisplatin, and 5-FU induction chemotherapy followed by chemoradiotherapy in locoregionally advanced nasopharyngeal cancer. Cancer chemotherapy and pharmacology. 2010;65(3):589–95. 10.1007/s00280-009-1152-0 . 19830427

[18] Lai SZ, Li WF, Chen L, Luo W, Chen YY, Liu LZ, et al How does intensity-modulated radiotherapy versus conventional two-dimensional radiotherapy influence the treatment results in nasopharyngeal carcinoma patients? International journal of radiation oncology, biology, physics. 2011;80(3):661–8. 10.1016/j.ijrobp.2010.03.024 . 20643517

[19] Lee AW, Lin JC, Ng WT. Current management of nasopharyngeal cancer. Seminars in radiation oncology. 2012;22(3):233–44. 10.1016/j.semradonc.2012.03.008 . 22687948

[20] Sun X, Su S, Chen C, Han F, Zhao C, Xiao W, et al Long-term outcomes of intensity-modulated radiotherapy for 868 patients with nasopharyngeal carcinoma: an analysis of survival and treatment toxicities. Radiotherapy and oncology: journal of the European Society for Therapeutic Radiology and Oncology. 2014;110(3):398–403. 10.1016/j.radonc.2013.10.020 . 24231245

[21] Wu F, Wang R, Lu H, Wei B, Feng G, Li G, et al Concurrent chemoradiotherapy in locoregionally advanced nasopharyngeal carcinoma: treatment outcomes of a prospective, multicentric clinical study. Radiotherapy and oncology: journal of the European Society for Therapeutic Radiology and Oncology. 2014;112(1):106–11. 10.1016/j.radonc.2014.05.005 . 24933452

[22] Sun X, Zeng L, Chen C, Huang Y, Han F, Xiao W, et al Comparing treatment outcomes of different chemotherapy sequences during intensity modulated radiotherapy for advanced N-stage nasopharyngeal carcinoma patients. Radiation oncology. 2013;8:265 10.1186/1748-717X-8-265 24219818 PMC3842780

[23] Lorch JH, Goloubeva O, Haddad RI, Cullen K, Sarlis N, Tishler R, et al Induction chemotherapy with cisplatin and fluorouracil alone or in combination with docetaxel in locally advanced squamous-cell cancer of the head and neck: long-term results of the TAX 324 randomised phase 3 trial. The Lancet Oncology. 2011;12(2):153–9. 10.1016/S1470-2045(10)70279-5 21233014 PMC4356902

[24] Leung TW, Tung SY, Sze WK, Wong FC, Yuen KK, Lui CM, et al Treatment results of 1070 patients with nasopharyngeal carcinoma: an analysis of survival and failure patterns. Head & neck. 2005;27(7):555–65. 10.1002/hed.20189 . 15880410

[25] Ma J, Mai HQ, Hong MH, Cui NJ, Lu TX, Lu LX, et al Is the 1997 AJCC staging system for nasopharyngeal carcinoma prognostically useful for Chinese patient populations? International journal of radiation oncology, biology, physics. 2001;50(5):1181–9. . 11483327 10.1016/s0360-3016(01)01537-1

[26] Shenouda G, Zhang Q, Ang KK, Machtay M, Parliament MB, Hershock D, et al Long-term results of radiation therapy oncology group 9903: a randomized phase 3 trial to assess the effect of erythropoietin on local-regional control in anemic patients treated with radiation therapy for squamous cell carcinoma of the head and neck. International journal of radiation oncology, biology, physics. 2015;91(5):907–15. 10.1016/j.ijrobp.2014.12.018 . 25670542 PMC4657552

[27] Kodaira T, Fuwa N, Tachibana H, Hidano S. Phase I study of S-1 and nedaplatin for patients with recurrence of head and neck cancer. Anticancer research. 2006;26(3B):2265–8. . 16821599

[28] Al-Sarraf M, LeBlanc M, Giri PG, Fu KK, Cooper J, Vuong T, et al Chemoradiotherapy versus radiotherapy in patients with advanced nasopharyngeal cancer: phase III randomized Intergroup study 0099. Journal of clinical oncology: official journal of the American Society of Clinical Oncology. 1998;16(4):1310–7. . 9552031 10.1200/JCO.1998.16.4.1310

[29] Lee AW, Tung SY, Chua DT, Ngan RK, Chappell R, Tung R, et al Randomized trial of radiotherapy plus concurrent-adjuvant chemotherapy vs radiotherapy alone for regionally advanced nasopharyngeal carcinoma. Journal of the National Cancer Institute. 2010;102(15):1188–98. 10.1093/jnci/djq258 . 20634482

