# Supplementary material for: Long-Term Results of Concurrent Chemoradiotherapy for Advanced N2-3 Stage Nasopharyngeal Carcinoma
Source: PLoS One. 2015 Sep 14;10(9):e0137383. doi: 10.1371/journal.pone.0137383 (PMC4569428; doi:10.1371/journal.pone.0137383)
Supplement: S1 Table — (DOCX) [file pone.0137383.s002.docx]

**S1 Table. Univariate prognostic factor analyses for various clinical endpoints**

| **Factor** | **N** | **OS**  **(5-yr)** | ***P* value** | **PFS**  **(5-yr)** | ***P* value** | **LRRFS**  **(5-yr)** | *P* **value** | **DMFS**  **(5-yr)** | ***P* value** |
| --- | --- | --- | --- | --- | --- | --- | --- | --- | --- |
| Gender | | | | | | | | | |
| Male | 91 | 79.4% | 0.826 | 65.8% | 0.038 | 71.3% | 0.769 | 73.8% | 0.018 |
| Female | 37 | 85.9% |  | 83.8% |  | 86.5% |  | 83.8% |  |
| Age (yr) | | | | | | | | | |
| ≤50 | 69 | 83.3% | 0.845 | 76.5% | 0.334 | 80.7% | 0.093 | 79.3% | 0.731 |
| ＞50 | 59 | 79.1% |  | 64.3% |  | 69.9% |  | 73.4% |  |
| AJCC staging | | | | | | | | | |
| Ⅲ | 76 | 90.8% | 0.001 | 82.5% | 0.001 | 88.2% | 0.076 | 85.1% | 0.002 |
| Ⅳ | 52 | 67.2% |  | 53.6% |  | 57.8 % |  | 63.9% |  |
| AJCC T classification | | | | | | | | | |
| T1-2 | 35 | 88.2% | 0.094 | 76.2% | 0.091 | 79.6% | 0.019 | 84.8% | 0.602 |
| T3-4 | 93 | 78.7% |  | 69.3% |  | 74.3% |  | 73.9% |  |
| AJCC N classification | | | | | | | | | |
| N2 | 103 | 85.0% | 0.053 | 75.2% | 0.034 | 80.3% | 0.538 | 80.0% | 0.002 |
| N3 | 25 | 66.4% |  | 53.5% |  | 57.0% |  | 62.5% |  |
| Radiation dose to primary site or metastatic LN (Gy) | | | | | | | | | |
| ≤70 | 25 | 84.0% | 0.113 | 72.0% | 0.756 | 80.0% | 0.531 | 76.0% | 0.334 |
| ＞70 | 103 | 80.6% |  | 70.8% |  | 74.7% |  | 76.8% |  |
| Adjuvant chemotherapy | | | | | | | | | |
| No | 10 | 60.0% | 0.873 | 59.6% | 0.660 | 89.9% | 0.487 | 60.0% | 0.166 |
| 1-2 cycles | 83 | 76.8% |  | 66.0% |  | 84.0% |  | 77.6% |  |
| 3-4 cycles | 35 | 82.3% |  | 77.4% |  | 90.1% |  | 90.5% |  |
